# Supplementary material for: Longevity and Neutralizing Capacity of IgG Antibodies against SARS-CoV-2 Generated by the Application of BNT162b2, AZD1222, Convidecia, Sputnik V, and CoronaVac Vaccines: a Cohort Study in the Mexican Population
Source: Microbiol Spectr. 2023 Jan 5;11(1):e02376-22. doi: 10.1128/spectrum.02376-22 (PMC9927513; doi:10.1128/spectrum.02376-22)
Supplement: Supplemental file 2 — Fig. S1 and S2 and Tables S1 and S2. Download spectrum.02376-22-s0002.pdf, PDF file, 0.4 MB [file spectrum.02376-22-s0002.pdf]

SUPPLEMENTAL MATERIAL FOR PUBLICATION

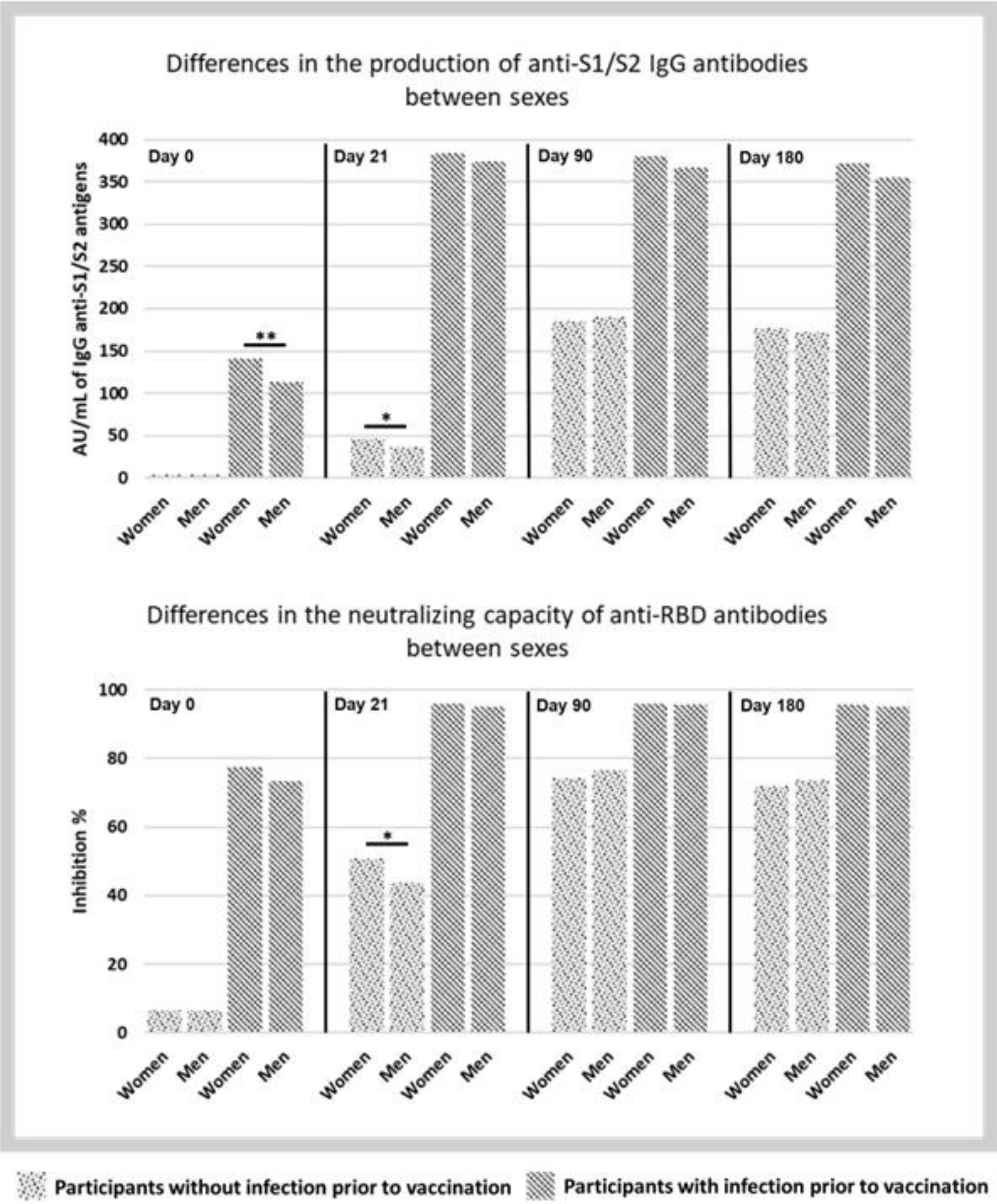

Fig. S1. Differences in the production of anti-S1/S2 IgG antibodies and the neutralizing capacity of anti-RBD antibodies between sexes.

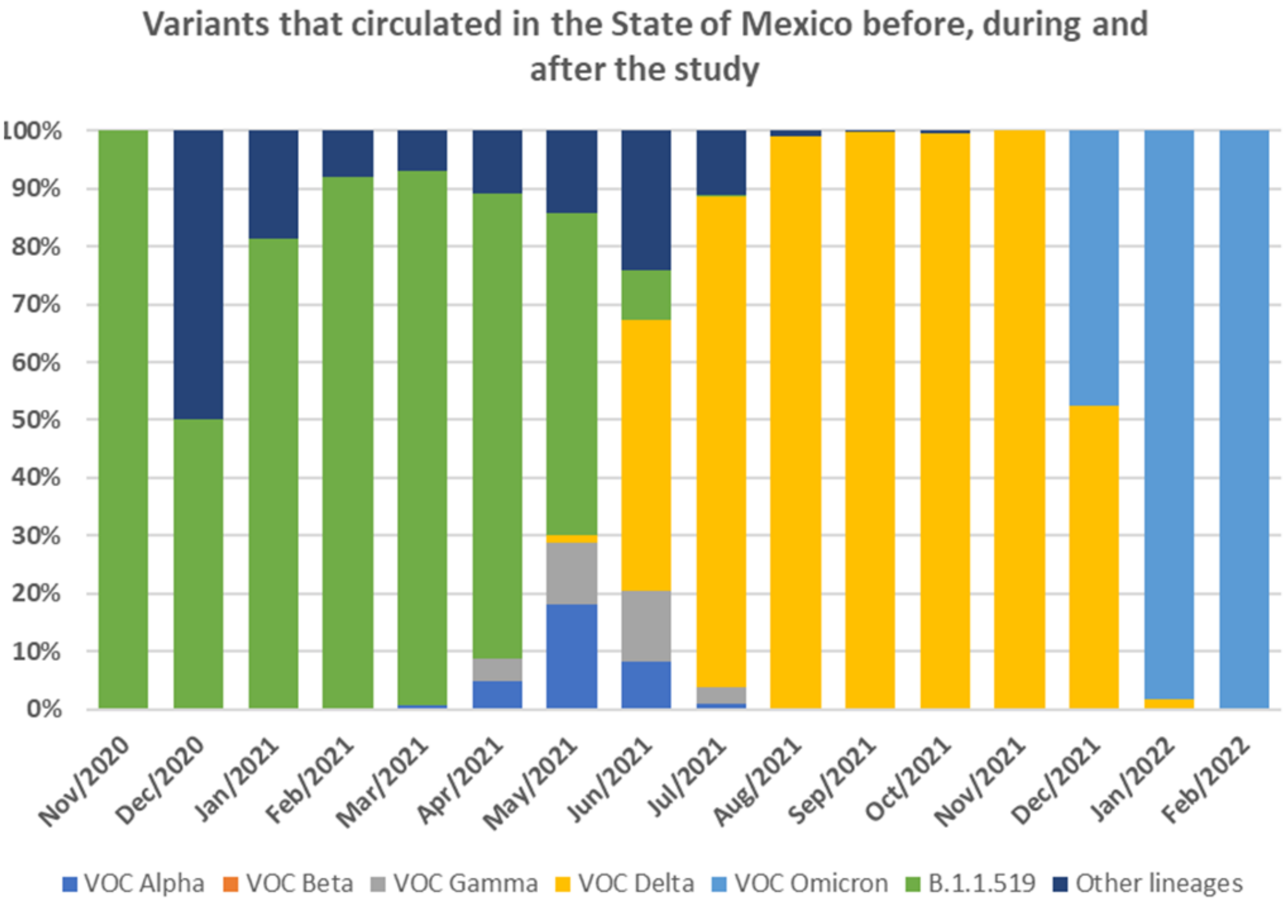

Fig. S2. Variants that circulated in the State of Mexico before, during and after the study. Before: Nov/2020-Apr/2021; during: May/2021-Jan/2022; after: Feb/2022.

Supplemental Table 1. Confidence intervals of the data shown in Fig. 7.

Antibodies IgG anti-S1/S2 (AU/mL)

VACCINE	Sample 1 mean (CI)	Sample 2 mean (CI)	Sample 3 mean (CI)	Sample 4 mean (CI)
Participants without previous infection				
**BNT162b2**	3.8 (3.7-3.9)	58.3 (47.8-68.8)	331.3 (299.2-363.4)	203.8 (163.0-244.6)
**AZD1222**	3.8 (3.7-3.9)	34.9 (26.4-43.5)	230.6 (206.9-254.3)	94.7 (56.0-133.4)
**Convidecia**	3.8 (3.7-3.9)	55.3 (47.9-62.7)	59.6 (34.6-84.6)	126.1 (93.0-159.2)
**Sputnik V**	3.9 (3.8-4.0)	42.8 (34.0-51.7)	269.9 (243.1-296.6)	249.6 (206.2-293.0)
**CoronaVac**	4.0 (3.9-4.2)	20.3 (9.9-30.7)	57.4 (26.4-88.5)	275.3 (228.9-321.8)
Participants with previous infection				
**BNT162b2**	167.5 (146.4-188.5)	400.0 (384.2-415.8)	400.0 (385.5-414.5)	400.0 (381.5-418.5)
**AZD1222**	161.8 (140.1-183.4)	400.0 (384.7-415.3)	400.0 (384.9-415.2)	400.0 (379.3-420.7)
**Convidecia**	115.1 (89.9-140.3)	400.0 (383.8-416.2)	400.0 (383.1-416.9)	400.0 (379.3-420.7)
**Sputnik V**	147.2 (123.6-170.8)	400.0 (381.9-418.1)	400.0 (383.3-416.7)	400.0 (379.0-421.1)
**CoronaVac**	79.7 (59.7-99.8)	309.1 (293.0-325.1)	282.5 (265.8-299.2)	231.4 (210.7-252.1)

Neutralizing capacity of antibodies against the RBD portion (% de inhibition)

VACCINE	Sample 1 mean (CI)	Sample 2 mean (CI)	Sample 3 mean (CI)	Sample 4 mean (CI)
Participants without previous infection				
**BNT162b2**	5.4 (4.1-6.7)	69.9 (61.7-78.1)	95.3 (88.7-102.0)	89.3 (81.8-96.9)
**AZD1222**	10.0 (9.0-11.0)	45.3 (38.7-51.9)	87.5 (82.6-92.4)	71.5 (65.1-78.0)
**Convidecia**	7.3 (6.4-8.3)	50.2 (44.5-56.0)	49.7 (45.0-54.4)	50.4 (44.4-56.4)
**Sputnik V**	4.5 (3.4-5.6)	47.1 (40.2-54.1)	94.5 (88.6-100.3)	88.5(80.0-97.0)
**CoronaVac**	5.8 (4.7-6.9)	32.2 (24.6-39.8)	62.4 (56.5-68.3)	81.6 (73.0-90.0)
Participants with previous infection				
**BNT162b2**	77.9 (73.5-82.3)	92.7 (91.4-94.0)	95.9 (95.5-96.3)	96.2 (95.3-97.1)
**AZD1222**	82.4 (77.9-87.0)	97.0 (95.8-98.4)	96.0 (95.6-96.5)	96.4 (95.4-97.3)
**Convidecia**	82.2 (77.0-87.5)	97.0 (95.6-98.3)	96.0 (95.5-96.5)	96.2 (95.3-97.1)
**Sputnik V**	73.3 (68.4-78.2)	97.2 (95.7-98.7)	96.8 (96.3-97.3)	95.9 (94.9-96.9)

**CoronaVac**	72.0 (68.0-76.0)	95.0 (93.7-96.5)	94.5 (94.0-95.0)	93.1 (92.1-94.2)

Supplemental Table 2. Analysis of the amount of anti-S1/S2 IgG antibodies and neutralizing capacity between the groups with and without comorbidity.

SAMPLE 2

PARTICIPANTS WITHOUT COVID-19 PRIOR TO VACCINATION

	Mean of IgG anti-S1/S2 antibodies (AU/mL)			Average percentage neutralization of anti-RBD antibodies		
	**YES**	**NO**	**p-value**	**YES**	**NO**	**p-value**
**DIABETES**	40.2	49.6	0.270	48.8	48.3	0.999
**OBESITY**	44.4	50.0	0.344	48.3	48.4	0.892
**ARTERIAL HYPERTENSION**	39.2	50.0	0.362	50.0	48.1	0.750
**IMMUNOSUPPRESSION**	51.6	48.6	0.469	42.6	48.5	0.529

PARTICIPANTS WITH COVID-19 PRIOR TO VACCINATION

	Mean of IgG anti-S1/S2 antibodies (AU/mL)			Average percentage neutralization of anti-RBD antibodies		
	**YES**	**NO**	**p-value**	**YES**	**NO**	**p-value**
**DIABETES**	390.1	370.1	0.231	91.9	92.0	**0.014**
**OBESITY**	372.1	372.9	0.860	90.8	92.7	0.082
**ARTERIAL HYPERTENSION**	396.2	367.9	**0.025**	92.5	91.9	**0.025**
**IMMUNOSUPPRESSION**	384.3	372.2	0.408	96.8	91.9	0.225

SAMPLE 3

PARTICIPANTS WITHOUT COVID-19 PRIOR TO VACCINATION

	Mean of IgG anti-S1/S2 antibodies (AU/mL)			Average percentage neutralization of anti-RBD antibodies		
	**YES**	**NO**	**p-value**	**YES**	**NO**	**p-value**
**DIABETES**	199.7	191.3	0.794	72.4	72.4	0.932
**OBESITY**	218.8	284.2	0.103	75.9	71.3	0.249
**ARTERIAL HYPERTENSION**	224.8	186.2	0.107	78.0	71.4	0.110
**IMMUNOSUPPRESSION**	228.5	190.9	0.816	61.7	72.7	0.732

PARTICIPANTS WITH COVID-19 PRIOR TO VACCINATION

	Mean of IgG anti-S1/S2 antibodies (AU/mL)			Average percentage neutralization of anti-RBD antibodies		
	**YES**	**NO**	**p-value**	**YES**	**NO**	**p-value**
**DIABETES**	399.6	368.4	0.083	89.8	93.0	0.894
**OBESITY**	372.2	272.3	0.810	92.3	92.8	0.217
**ARTERIAL HYPERTENSION**	390.6	368.3	**0.027**	91.9	92.8	0.086
**IMMUNOSUPPRESSION**	363.0	372.7	0.637	94.3	92.5	0.551

SAMPLE 4

PARTICIPANTS WITHOUT COVID-19 PRIOR TO VACCINATION						
	Mean of IgG anti-S1/S2 antibodies (AU/mL)			Average percentage neutralization of anti-RBD antibodies		
	SÍ	NO	p-value	SÍ	NO	p-value
DIABETES	159.9	188.2	0.291	64.1	71.0	0.161
OBESITY	180.1	186.9	0.679	67.8	71.2	0.617
ARTERIAL HYPERTENSION	151.6	192.1	0.172	65.7	71.2	0.068
IMMUNOSUPPRESSION	251.6	182.6	0.507	66.1	70.4	0.924

PARTICIPANTS WITH COVID-19 PRIOR TO VACCINATION						
	Mean of IgG anti-S1/S2 antibodies (AU/mL)			Average percentage neutralization of anti-RBD antibodies		
	YES	NO	p-value	YES	NO	p-value
DIABETES	383.7	358.1	0.182	89.5	93.1	0.801
OBESITY	341.2	374.0	0.084	90.7	93.8	0.463
ARTERIAL HYPERTENSION	376.5	358.2	0.179	93.0	92.5	0.642
IMMUNOSUPPRESSION	400.0	360.1	0.240	94.3	92.6	0.181
